# Trends and Influencing Factors in Temporal Psychological Well-Being of Adolescents: Evidence from a Longitudinal Study

**DOI:** 10.3390/bs16060889

**Published:** 2026-06-01

**Authors:** Yuanchao Hu, Liqiang Zhang, Tongshuang Yuan, Yujie Cui, Kai Liu, Fangfang Ding, Yaning Su, Chaofan Zhang, Liru Pan, Chengbin Zheng, Songli Mei

**Affiliations:** School of Public Health, Jilin University, Changchun 130021, China

**Keywords:** temporal psychological well-being, latent growth curve model, adolescents, developmental trajectories

## Abstract

Temporal psychological well-being (subjective well-being, future confidence and life satisfaction) is an important indicator of the level of mental health and well-being. During adolescence, the dynamic development of their psychological well-being is more susceptible to multiple factors. However, the trends and multidimensional influences on adolescents’ temporal psychological well-being, as well as their co-development with the relationship between interpersonal relationships and temporal psychological well-being, are not known. Therefore, 568 adolescents (M_age_ ± SD = 17.41 ± 1.14) aged 16 to 19 years were selected for this study using longitudinal data from the 2018, 2020, and 2022 China Family Panel Studies (CFPS). Analyses were conducted using the latent growth curve model (LGCM). It was found that adolescents’ subjective well-being, future confidence, and life satisfaction showed a significant linear decline. Health status, academic pressure, and parent–child relationships were significantly associated with the initial level and developmental trajectory of temporal psychological well-being. In addition, we observed co-development patterns between interpersonal relationships and adolescents’ temporal psychological well-being, such that parallel changes in interpersonal relationships coincided with changes in well-being over time, and vice versa. This study reveals the dynamic patterns of changes in adolescents’ temporal psychological well-being and their co-development with relationships, providing empirical evidence for targeted interventions.

## 1. Introduction

Adolescence represents a critical transitional stage from childhood to adulthood, during which psychological well-being not only shapes current quality of life and mental health but also exerts profound and lasting influences on later developmental outcomes (Kapadia, 2024; Sawyer et al., 2012). Psychological challenges are common among adolescents, which may manifest as interpersonal conflicts and academic pressure, among others. If these psychological issues remain unresolved over the long-term, they may hinder adolescents’ potential development and negatively impact their psychological resilience building. Thus, delving into the developmental trends and influencing factors of psychological well-being in adolescents is of vital theoretical and practical importance for promoting their healthy growth and all-round development (Sawyer et al., 2012).

According to the two-factor model, mental health is made up of the dynamic balance of positive and negative emotions (Suldo & Shaffer, 2008). This theoretical framework expands the conventional boundaries of understanding mental health and overcomes the limitation of prior studies, which only focus on negative emotions like anxiety and depression. This study emphasizes the key positive dimension of psychological well-being. Psychological well-being, reflecting an individual’s comprehensive experience of life satisfaction, positive emotions, and good psychological functioning, is a key indicator of mental health and happiness levels (Volkova & Sorokoumova, 2024). It is central to psychological development and quality of life research across different age groups. However, psychological well-being is not static but has a dynamic temporal dimension. According to the time perspective (TP) theory, TP, as a fundamental dimension of psychological time, permeates the cognitive process of dividing human experiences into past, present, and future time frames. It concerns both an individual’s current psychological well-being and their recollection and evaluation of the past as well as the expectations and outlook for the future (Sailer et al., 2014; Zimbardo & Boyd, 1999). Based on this, this study introduces the concept of temporal psychological well-being. From this perspective, it emphasizes individuals’ perception and experience of their own psychological well-being across different time dimensions (i.e., the past, present, and future).

Despite the theoretical appeal of temporal psychological well-being, several critical issues remain unresolved in the literature. First, operational definitions of this construct vary considerably across studies: some scholars treat it as a unidimensional construct reflecting overall time-oriented well-being (Drake et al., 2008), while others conceptualize it as comprising distinct dimensions corresponding to past, present, and future time frames (Durayappah, 2011; Sailer et al., 2014). This definitional inconsistency complicates cross-study comparisons and theoretical advancement. Second, existing research on temporal psychological well-being has predominantly focused on Western cultural contexts, raising questions about its cross-cultural applicability. Cultural differences in time orientation—such as the greater emphasis on future planning in East Asian cultures compared to present-focused orientation in some Western contexts—may fundamentally alter the structure and correlates of temporal well-being (Zimbardo & Boyd, 1999). Third, the integration between time perspective theory and subjective well-being research remains insufficient; while both fields have developed extensively in isolation, a unified theoretical framework that bridges temporal cognition and well-being assessment is still lacking. These gaps highlight the need for longitudinal research in diverse cultural settings that examines temporal psychological well-being as a dynamic, multidimensional construct. This study employs a theory-based multidimensional approach by examining the developmental trajectories of temporal psychological well-being among Chinese adolescents and its ecological factors, to offer new insights for related research.

This study selected life satisfaction, subjective well-being, and future confidence, three positive emotions closely related to time perspective, as dependent variables (Drake et al., 2008; Garcia et al., 2011; Parrello et al., 2024). Drawing on the 3P model, subjective well-being comprises the three time dimensions of the past, present, and future, that is, it reflects an individual’s subjective perception and overall emotional evaluation of life across these three dimensions; future confidence refers to an individual’s confidence and optimism about future life, a key component of temporal psychological well-being; and life satisfaction is a person’s cognitive evaluation of their overall life, covering the past, present, and future (Diener, 1984; Durayappah, 2011; Joutsenniemi et al., 2013; Pan et al., 2023; R. N. Wang et al., 2022). Temporal psychological well-being significantly impacts their social adaptability, academic achievement, and psychological resilience. Research shows that higher subjective well-being can enhance positive emotional experiences in adolescents, promoting their success in academics, social life, and careers (Putwain et al., 2020). A high level of future confidence can significantly reduce the severity of depressive symptoms (R. N. Wang et al., 2022). Life satisfaction plays a key role in predicting student engagement and academic achievement (Heffner & Antaramian, 2016).

This multi-temporal perspective provides richer information and deeper insights into the developmental process of psychological well-being in adolescents, which helps reveal the dynamic patterns of psychological well-being over time, its influencing factors, and its complex relationship with interpersonal relationships. However, there is still a lack of clear research on the future trajectory of psychological well-being in adolescents under a balanced time frame. To fill this research gap, this study focused on the future trends and influencing factors of temporal psychological well-being in Chinese adolescents transitioning from adolescence to early and mid-adulthood.

### 1.1. The Trajectory of Adolescent Temporal Psychological Well-Being

Previous studies have found that the developmental trends of subjective well-being, future confidence, and life satisfaction in adolescence show significant dynamism, but no consistent conclusions have been reached. Many longitudinal studies indicate that these factors generally decline during adolescence (Herke et al., 2019; Orben et al., 2022). This may be related to the multiple pressures that adolescents face during this stage, including gender issues, academic burden, peer pressure, and future uncertainties (Jeong et al., 2018). However, some studies suggest that in specific cultural contexts, subjective well-being, future confidence, and life satisfaction may rebound during certain phases (Blanchflower & Oswald, 2008). For instance, in Finland, life satisfaction among adolescents increases during educational transitions (Salmela-Aro & Tuominen-Soini, 2010). Other research shows that from adolescence through early and mid-adulthood, life satisfaction gradually declines before starting to rise in later adulthood (Cheng et al., 2017; Piper, 2015). These findings indicate that the changes in subjective well-being, future confidence, and life satisfaction are influenced not only by individual development but also by cultural background and social environment.

### 1.2. Relationship Between Covariates and Adolescent Temporal Psychological Well-Being

Drawing on Bronfenbrenner’s Bioecological Model (Bronfenbrenner, 1979), the development of adolescents is viewed as a dynamic, multi-level ecosystem involving complex interactions between individual characteristics and family, community and environmental factors (Ettinger et al., 2022; Y. Q. Hu et al., 2023). Within this framework, we organize the predictors of temporal psychological well-being according to their ecological level of influence. At the microsystem level, individual and family factors exert the most direct effects on adolescent development. Firstly, under the interactive effects of gender and time, some studies indicate significant gender differences in adolescent mental health (Yoon et al., 2023). Health status is a vital foundation of psychological well-being, as physical health directly impacts adolescents’ emotional experiences, quality of life, and psychological functioning (Wolf & Schmitz, 2024). Good health enables adolescents to participate actively in various activities, boosting their confidence and happiness. In contrast, health problems can cause physical discomfort and psychological stress, reducing psychological well-being levels. Secondly, academic pressure is a significant stressor in adolescent development (Steare et al., 2023). Extensive research shows a significant negative correlation between academic pressure and adolescents’ temporal psychological well-being (Crisp et al., 2025). Excessive academic burden can lead to anxiety, depression, and other negative emotions, lowering life satisfaction and happiness (C. H. Wang et al., 2025). Parental relationships are a key component of adolescents’ social support system. Good parent–child relationships provide emotional support and a sense of security, promoting the development of positive psychological qualities and enhancing psychological well-being (Ying & Han, 2008). Conversely, tense or conflictual parent–child relationships can adversely affect adolescents’ temporal psychological well-being (Ding et al., 2025). At the mesosystem level, Internet use serves as a bridge connecting school and family environments, exerting a dual impact on temporal psychological well-being (Montag et al., 2024). On the one hand, moderate Internet use can meet adolescents’ social needs, provide information, and offer relaxation, thereby improving temporal psychological well-being (Balamurali, 2025). On the other hand, excessive screen time or exposure to harmful online content can lead to Internet addiction, depression, anxiety, and other psychological issues, damaging temporal psychological well-being (Santos et al., 2023). The impact of these factors on the developmental trajectory of psychological well-being in the adolescent stage is unclear.

Based on ecosystem theory and a comprehensive literature review, this study selects gender, health status, academic pressure, parent–child relationships, and Internet use as the main factors influencing adolescents’ temporal psychological well-being.

### 1.3. Interpersonal Relationships and Adolescents’ Temporal Psychological Well-Being

Interpersonal relationships reflect adolescents’ social status and popularity in peer groups. Good interpersonal relationships can provide adolescents with more social support and positive interactive experiences, thereby enhancing their temporal psychological well-being (Li et al., 2020). In contrast, poor interpersonal relationships or peer rejection can lead to loneliness, inferiority, and other negative emotions, negatively impacting temporal psychological well-being (Huang et al., 2022). As adolescents grow older, they tend to devote more time to interacting with peers, especially in peer groups (Jiao et al., 2017). According to group socialization theory, peer groups shape adolescents’ social development more than parents do (Harris, 2000). Early adolescence is a stage of significant enhancement in socio-emotional sensitivity (Laursen & Veenstra, 2021). During this period, individuals’ attention to peers peaks, triggering strong self-conscious emotions and increasing brain activity related to social feedback (Somerville et al., 2013). As adolescents approach this critical transitional period, their interactions with peers become more frequent and in-depth (Cairns et al., 1995). They are extremely sensitive and reactive to peer evaluations and feedback. Thus, interpersonal relationships are highly significant for adolescent development at this stage.

Existing research indicates a significant association between interpersonal relationships and adolescents’ temporal psychological well-being (Jiao et al., 2017; Zambrano et al., 2024). However, few studies have examined the developmental trajectories of these two variables, including their patterns of co-development. Therefore, this study incorporates interpersonal relationships as a time-varying variable to investigate the longitudinal developmental relationship between interpersonal relationships and adolescents’ temporal psychological well-being and its potential mechanisms.

### 1.4. Present Study

Despite existing research on adolescent psychological well-being trends and influences, the literature mainly focuses on single well-being indicators and limited factors, lacking an ecological systems theory approach. This makes it hard to uncover long-term well-being trends and complex influence mechanisms. Also, prior studies often emphasized immediate interpersonal relationship effects, overlooking their dynamic interplay with adolescents’ temporal psychological well-being. To address this, this study applies latent growth curve model to thoroughly examine the development trend of adolescents’ temporal psychological well-being and explore the intricate influence mechanisms behind it. Specifically, this study addresses the following research questions and hypotheses:

Research Question 1: What developmental trends do subjective well-being, future confidence, and life satisfaction exhibit during ages 16–19? Hypothesis 1a: Subjective well-being shows a linear downward trend during adolescence (Herke et al., 2019). Hypothesis 1b: Future confidence shows a linear downward trend during adolescence (R. N. Wang et al., 2022). Hypothesis 1c: Life satisfaction shows a linear downward trend during adolescence (H. Kim et al., 2020).

Research Question 2: How are health status, academic pressure, parent–child relationships, Internet use, and gender associated with the initial levels and developmental trajectories of adolescent temporal psychological well-being? Hypothesis 2a: Health status is positively associated with initial levels of temporal psychological well-being. Hypothesis 2b: Academic pressure is negatively associated with initial levels and slopes of temporal psychological well-being (Steare et al., 2023). Hypothesis 2c: Parent–child relationships are positively associated with initial levels of temporal psychological well-being (Ying & Han, 2008).

Research Question 3: Do interpersonal relationships and adolescent temporal psychological well-being co-develop over time? Hypothesis 3a: Interpersonal relationships are positively associated with developmental trajectories of subjective well-being, future confidence, and life satisfaction. Hypothesis 3b: Subjective well-being, future confidence, and life satisfaction are positively associated with developmental trajectories of interpersonal relationships.

## 2. Methods

### 2.1. Data and Sample

This study utilized data from the 2018, 2020, and 2022 waves of the China Family Panel Studies (CFPS), a nationally representative longitudinal survey designed and implemented by the Institute of Social Science Survey at Peking University. The CFPS collects data at the community, family, and individual levels, covering 25 provinces/municipalities/autonomous regions (excluding Inner Mongolia, Xinjiang, Tibet, Hainan, Ningxia, and Qinghai) (Xie & Hu, 2014). The survey was approved by the Peking University Ethics Committee (No. IRB00001052-14010). Prior to participation, all respondents provided informed consent. All personal information was de-identified, and this study constitutes secondary data analysis of anonymized CFPS data. To reflect the latest changes in adolescents’ temporal psychological well-being, this study selected the latest three waves of survey data. After matching the 2018 baseline individual response database with the 2020 and 2022 databases and excluding cases with missing information, a final sample of 568 adolescents aged 16–19 (49.8% male, M_age_ ± SD = 17.41 ± 1.14) was obtained.

### 2.2. Measures

#### 2.2.1. Subjective Well-Being

Subjective well-being was tested with one question, “How happy do you consider yourself to be?”. The score is based on a 0–10 scale, with 0 indicating “lowest happiness” and 10 indicating “highest happiness”. Higher scores represent better subjective well-being. This format is consistent with widely used single-item happiness measures in well-being research (Fordyce, 1988). The single-item happiness measures have been extensively validated in adolescent populations (Shi et al., 2022; Wei & Zhang, 2025).

#### 2.2.2. Future Confidence

Future confidence was tested with one question, “How confident are you in your future?”. The score is based on a 1–5 Likert scale, where 1 represents “not confident at all” and 5 represents “very confident”. Higher scores represent greater optimism about the future. This item was adapted from Joutsenniemi et al. (2013), who used this approach in a large-scale Finnish health behavior survey (N = 101,257). The item captures an individual’s generalized optimism about future life, which is a key component of temporal psychological well-being (Joutsenniemi et al., 2013).

#### 2.2.3. Life Satisfaction

Life satisfaction was tested with one question, “How satisfied are you with your life?”. The score is based on a 1–5 scale, where 1 indicates “very dissatisfied” and 5 indicates “very satisfied”. Higher scores represent greater life satisfaction. Single-item life satisfaction measures have been shown to perform as well as the Satisfaction with Life Scale in adolescent samples, producing equivalent patterns of associations with theoretically relevant variables (Jovanovic, 2016). This item has also been used to assess life satisfaction among adolescents in several studies (Chen et al., 2021).

#### 2.2.4. Interpersonal Relationships

Interpersonal relationships were tested with one question, “How good do you consider your interpersonal relationships to be?”. The score is based on a 0–10 scale, where 0 represents “worst” and 10 represents “best”. Higher scores represent better interpersonal relationships. This item has been consistently used across all waves of the CFPS survey, demonstrating good longitudinal consistency and stability in measuring adolescents’ self-perceived interpersonal relationship quality (Xie & Hu, 2014).

#### 2.2.5. Basic Demographic Variables

This study includes multiple basic demographic factors of adolescents, such as age, gender, health status, academic pressure, parent–child relationships, and Internet use. Academic pressure was tested with one question, “How much pressure do you feel in your studies?”. The score is based on a 1–5 Likert scale, where 1 represents “no pressure” and 5 represents “a lot of pressure”. Higher scores indicate greater pressure. Relationships with mother and father were each tested with one question, “How would you describe your relationship with your mother/father over the past six months?”. The score is based on a scale of 1–5 (7 indicates deceased and is not read aloud). Internet use was tested with one question, “Do you use mobile devices (e.g., smartphones, tablets) to access the Internet?”, where 1 represents “yes” and 2 represents “no”. Health status was tested with one question, “How would you rate your health?”. The score is based on a 1–5 scale, where 1 represents “very healthy”, 2 represents “healthy”, 3 represents “fairly healthy”, 4 represents “average”, and 5 represents “unhealthy”.

### 2.3. Data Analysis Strategy

This study first conducted descriptive statistical analysis on variables such as health status, academic pressure, relationships with father and mother, Internet use, gender, subjective well-being, future confidence, and life satisfaction. Pearson correlation coefficients were used to examine the relationships between variables at different measurement points. Then, latent growth curve model (LGCM) was adopted to investigate the developmental trajectories and synchronous changing patterns of the target variables. Model fit was evaluated using comparative fit index (CFI), root mean square error of approximation (RMSEA), SRMR (standardized root mean square residual), and Tucker–Lewis index (TLI). A model was considered to have good fit when the CFI and TLI were greater than 0.90, and the RMSEA and SRMR were less than 0.08 (L. T. Hu & Bentler, 1999). The specific analysis steps were as follows. First, unconditional LGCMs were constructed for the three measurements of subjective well-being, future confidence, and life satisfaction without controlling for covariates. The intercept represented the initial level, and the slope represented the change trajectory, examining the developmental trends of the three variables. Second, conditional models for the developmental trends of subjective well-being, future confidence, and life satisfaction were constructed, examining the associations of each covariate with these variables. Third, parallel growth models were constructed for interpersonal relationships and the three psychological well-being variables. Data analysis was performed using SPSS 24.0 and Mplus 7.0.

## 3. Results

### 3.1. Descriptive and Correlation Analysis

**Figure 1:**
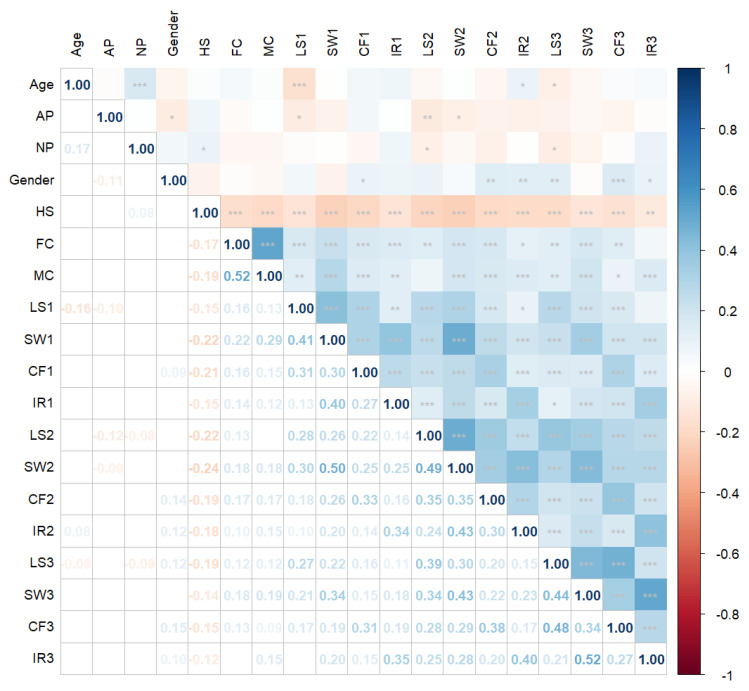
Correlation coefficient matrix of variables. Note: LS 1 = life satisfaction at time 1; LS 2 = life satisfaction at time 2; LS 3 = life satisfaction at time 3; CF 1 = future confidence at time 1; CF 2 = future confidence at time 2; CF 3 = future confidence at time 3; SW 1 = subjective well-being at time 1; SW 2 = subjective well-being at time 2; SW 3 = subjective well-being at time 3; IR 1 = interpersonal relationship at time 1; IR 2 = interpersonal relationship at time 2; IR 3 = interpersonal relationship at time 3; AP = academic pressure; NP = network participation; HS = health status; FC = father–child relationship; MC = mother–child relationship. * *p* < 0.05, ** *p* < 0.01, *** *p* < 0.001.

Figure 1 presents the relationships among variables such as subjective well-being, future confidence, life satisfaction, interpersonal relationships, and other categories across the three time points. The color intensity of each cell in Figure 1 represents the absolute value of the correlation coefficient, with darker colors indicating stronger correlations. Blue cells indicate positive correlations, while red cells indicate negative correlations.

Correlation analysis results indicate that at T1, father–child relationship and mother–child relationship were positively correlated with life satisfaction (*r* = 0.16, *p* < 0.001; *r* = 0.13, *p* < 0.01), while health status and academic pressure were negatively correlated with life satisfaction (*r* = −0.15, *p* < 0.001; *r* = −0.10, *p* < 0.05). Health status was negatively correlated with future confidence (*r* = −0.21, *p* < 0.001), whereas father–child relationship, mother–child relationship, and gender were positively correlated with future confidence (*r* = 0.16, *p* < 0.001; *r* = 0.15, *p* < 0.001; *r* = 0.09, *p* < 0.05). Father–child relationship and mother–child relationship were positively correlated with subjective well-being (*r* = 0.22, *p* < 0.001; *r* = 0.29, *p* < 0.001), and health status was negatively correlated with subjective well-being (*r* = −0.22, *p* < 0.001). The correlations of the variables at time points T2 and T3 are shown in Figure 1. The descriptive characteristics of the study participants are presented in Table A1.

### 3.2. Trends in Subjective Well-Being, Future Confidence, and Life Satisfaction

As shown in Table 1, the linear LGCM demonstrated good fit for subjective well-being (χ^2^ = 1.09, *CFI* = 1.00, *TLI* = 1.00, *RMSEA* = 0.01, *SRMR* = 0.01), future confidence (χ^2^ = 0.00, *CFI* = 1.00, *TLI* = 1.02, *RMSEA* = 0.00, *SRMR* = 0.00), life satisfaction (χ^2^ = 3.23, *CFI* = 0.99, *TLI* = 0.96, *RMSEA* = 0.06, *SRMR* = 0.02), and interpersonal relationships (χ^2^ = 0.01, *CFI* = 1.00, *TLI* = 1.01, *RMSEA* = 0.00, *SRMR* = 0.00). Results of the linear unconditional model for subjective well-being, future confidence, and life satisfaction indicated that subjective well-being had an intercept mean of 7.93 (*p* < 0.001) and variance of 1.60 (*p* < 0.001), showing significant individual differences in initial levels. The slope mean of −0.12 (*p* < 0.01) and variance of 0.25 (*p* < 0.01) indicated a linear downward trend in subjective well-being over the three measurements, with individual differences in the rate of decline. Future confidence had an intercept mean of 4.04 (*p* < 0.001) and variance of 0.18 (*p* < 0.001), indicating significant individual differences in initial levels. The slope mean of −0.08 (*p* < 0.001) and variance of 0.02 (*p* > 0.05) showed a linear downward trend in future confidence over the three measurements, with no significant individual differences in the rate of decline. Life satisfaction had an intercept mean of 3.94 (*p* < 0.001) and variance of 0.16 (*p* < 0.01), indicating significant individual differences in initial levels. The slope mean of −0.07 (*p* < 0.01) and variance of 0.04 (*p* > 0.05) indicated a linear downward trend in life satisfaction over the three measurements. Interpersonal relationships had an intercept mean of 6.99 (*p* < 0.001) and variance of 0.81 (*p* < 0.001), showing significant individual differences in initial levels. These results are consistent with Hypotheses 1a–c, demonstrating significant linear declines in all three indicators of temporal psychological well-being during adolescence.

### 3.3. Effects of Health Status, Academic Pressure, Parental Relationships, Internet Use, and Gender on Subjective Well-Being, Future Confidence, and Life Satisfaction

In order to explore whether there are differences in the developmental trajectories of health status, academic pressure, parent–child relationships, network participation, and gender on subjective well-being, future confidence, and life satisfaction, a conditional model was built by incorporating time-invariant covariates in a linear unconditional model.

**Figure 2:**
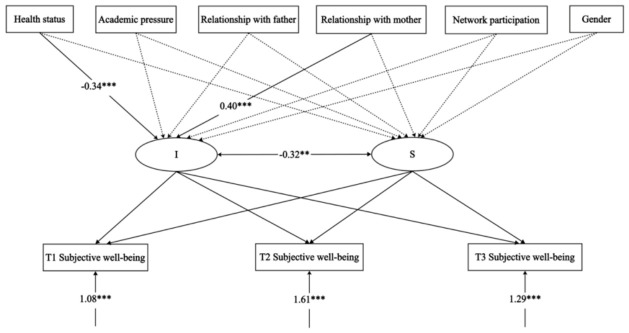
Conditional model of subjective well-being. Note: ** *p* < 0.01, *** *p* < 0.001.

**Figure 3:**
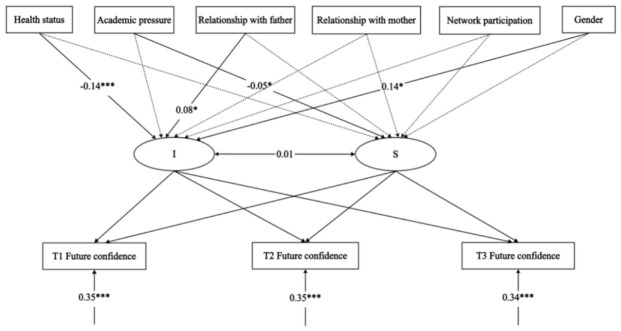
Conditional model of future confidence. Note: * *p* < 0.05, *** *p* < 0.001.

**Figure 4:**
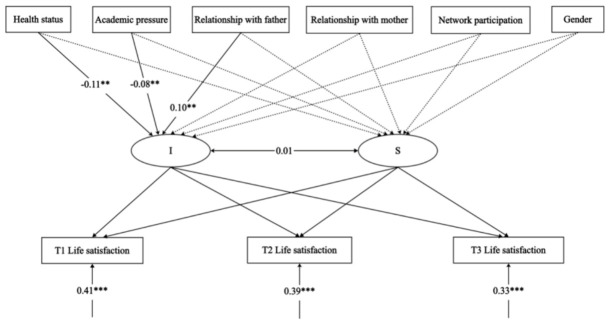
Conditional model of life satisfaction. Note: ** *p* < 0.01, *** *p* < 0.001.

As shown in Table A2, the three conditional models exhibited good fit. Figure 2 presents the conditional model for subjective well-being. Health status was negatively associated with the intercept of subjective well-being (*B* = −0.34, *p* < 0.001), and the mother–child relationship was positively associated with the intercept of subjective well-being (*B* = 0.40, *p* < 0.001). This indicates that individuals with better health status and more harmonious relationships with their mothers reported higher initial levels of subjective well-being. Figure 3 shows the conditional model for future confidence. Health status was negatively associated with the intercept of future confidence (*B* = −0.14, *p* < 0.001), the father–child relationship was positively associated with the intercept of future confidence (*B* = 0.08, *p* < 0.05), gender was positively associated with the intercept of future confidence (*B* = 0.14, *p* < 0.05), and academic pressure was negatively associated with the slope of future confidence (*B* = −0.05, *p* < 0.05). This suggests that individuals with better health status, more harmonious relationships with their fathers and male gender reported higher initial levels of future confidence, while those with greater academic pressure showed a steeper decline in future confidence. Figure 4 presents the conditional model for life satisfaction. Health status was negatively associated with the intercept of life satisfaction (*B* = −0.11, *p* < 0.01), academic pressure was negatively associated with the intercept of life satisfaction (*B* = −0.08, *p* < 0.01), and the father–child relationship was positively associated with the intercept of life satisfaction (*B* = 0.10, *p* < 0.01). This indicates that individuals with better health status, lower academic pressure, and more harmonious relationships with their fathers reported higher initial levels of life satisfaction. These results support Hypotheses 2a–c, demonstrating that health status, academic pressure, and parent–child relationships are significantly associated with the initial levels and developmental trajectories of temporal psychological well-being indicators.

### 3.4. Effects of Interpersonal Relationships on the Developmental Trajectories of Subjective Well-Being, Future Confidence, and Life Satisfaction

To systematically explore the co-development of interpersonal relationships and the developmental trajectories of subjective well-being, future confidence, and life satisfaction and further examine the concurrent associations between these variables and interpersonal relationships, parallel growth models were constructed. These models examined the associations between the intercept and slope of interpersonal relationships and the linear growth of subjective well-being, future confidence, and life satisfaction, and vice versa. As shown in Table A3, the six models demonstrated good fit.

**Figure 5:**
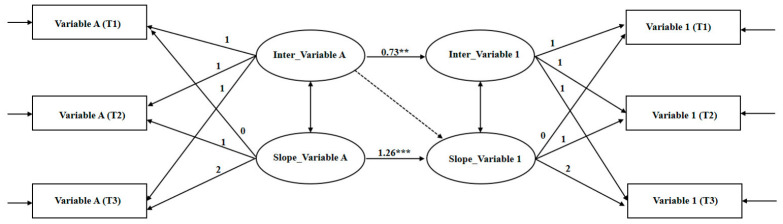
Parallel growth model of adolescents’ interpersonal relationships and subjective well-being (control variables omitted). Note: Variable A is interpersonal relationship, Variable 1 is subjective well-being; ** *p* < 0.01, *** *p* < 0.001; The numbers 0, 1, and 2 on the arrows from the slope factors to the observed variables represent the linear time scores (factor loadings) for Time 1, Time 2, and Time 3, respectively. The intercept factors have loadings fixed to 1 on all time points, representing the initial status.

**Figure A1:**
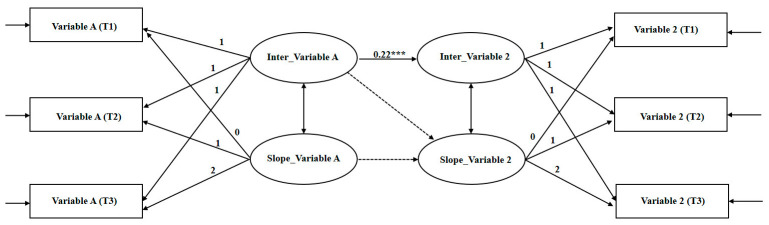
Parallel growth model of adolescents’ interpersonal relationships and future confidence (control variables omitted). Note: Variable A is interpersonal relationships, Variable 2 is future confidence; *** *p* < 0.001; The numbers 0, 1, and 2 on the arrows from the slope factors to the observed variables represent the linear time scores (factor loadings) for Time 1, Time 2, and Time 3, respectively. The intercept factors have loadings fixed to 1 on all time points, representing the initial status.

**Figure A2:**
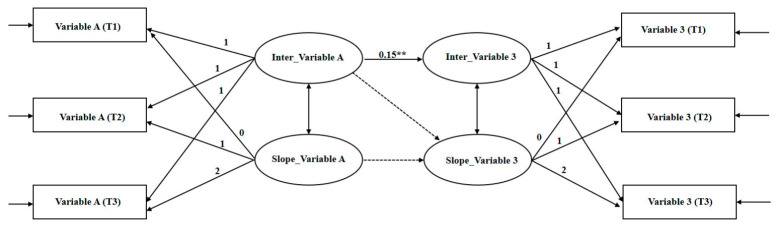
Parallel growth model of adolescents’ interpersonal relationships and life satisfaction (control variables omitted). Note: Variable A is interpersonal relationships, Variable 3 is life satisfaction; ** *p* < 0.01; The numbers 0, 1, and 2 on the arrows from the slope factors to the observed variables represent the linear time scores (factor loadings) for Time 1, Time 2, and Time 3, respectively. The intercept factors have loadings fixed to 1 on all time points, representing the initial status.

**Figure A3:**
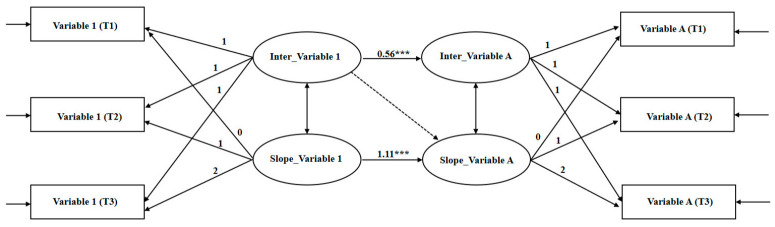
Parallel growth model of adolescents’ subjective well-being and interpersonal relationships (control variables omitted). Note: Variable A is interpersonal relationships, Variable 1 is subjective well-being; *** *p* < 0.001; The numbers 0, 1, and 2 on the arrows from the slope factors to the observed variables represent the linear time scores (factor loadings) for Time 1, Time 2, and Time 3, respectively. The intercept factors have loadings fixed to 1 on all time points, representing the initial status.

**Figure A4:**
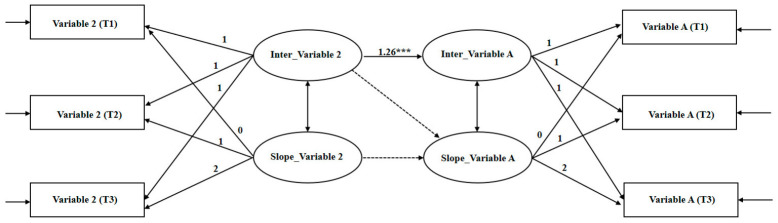
Parallel growth model of adolescents’ future confidence and interpersonal relationships (control variables omitted). Note: Variable A is interpersonal relationships, Variable 2 is future confidence; *** *p* < 0.001; The numbers 0, 1, and 2 on the arrows from the slope factors to the observed variables represent the linear time scores (factor loadings) for Time 1, Time 2, and Time 3, respectively. The intercept factors have loadings fixed to 1 on all time points, representing the initial status.

**Figure A5:**
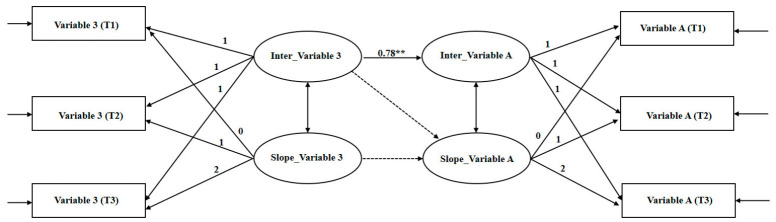
Parallel growth model of adolescents’ life satisfaction and interpersonal relationships (control variables omitted). Note: Variable A is interpersonal relationships, Variable 3 is life satisfaction; ** *p* < 0.01; The numbers 0, 1, and 2 on the arrows from the slope factors to the observed variables represent the linear time scores (factor loadings) for Time 1, Time 2, and Time 3, respectively. The intercept factors have loadings fixed to 1 on all time points, representing the initial status.

In the parallel growth model of interpersonal relationships and subjective well-being (see Figure 5), the regression coefficient of interpersonal relationship intercept on subjective well-being intercept was significant (*B* = 0.73, *SE* = 0.10, *p* < 0.01). This suggests that adolescents with higher initial levels of interpersonal relationships also reported higher initial levels of subjective well-being. Additionally, the slope of interpersonal relationships was positively associated with the slope of subjective well-being (*B* = 1.26, *SE* = 0.27, *p* < 0.001), suggesting that over time, adolescents with better-developing interpersonal relationships showed a more significant increase in subjective well-being. In the parallel growth model of interpersonal relationships and future confidence (see Appendix A Figure A1), the regression coefficient between interpersonal relationship intercept and future confidence intercept was significant (*B* = 0.22, *SE* = 0.04, *p* < 0.001), suggesting that adolescents with higher initial levels of interpersonal relationships also reported higher initial levels of future confidence. In the parallel growth model of interpersonal relationships and life satisfaction (see Appendix A Figure A2), the regression coefficient between interpersonal relationship intercept and life satisfaction intercept was significant (*B* = 0.15, *SE* = 0.05, *p* < 0.01), suggesting that adolescents with higher initial levels of interpersonal relationships also reported higher initial levels of life satisfaction. In the parallel growth model of subjective well-being and interpersonal relationships (see Appendix A Figure A3), the regression coefficient between subjective well-being intercept and interpersonal relationship intercept was significant (*B* = 0.56, *SE* = 0.07, *p* < 0.001), suggesting that adolescents with higher initial levels of subjective well-being also reported higher initial levels of interpersonal relationships. Furthermore, the slope of subjective well-being was positively associated with the slope of interpersonal relationships (*B* = 1.11, *SE* = 0.21, *p* < 0.001), suggesting that over time, adolescents with faster-growing subjective well-being also showed more significant development in interpersonal relationships. In the parallel growth model of future confidence and interpersonal relationships (see Appendix A Figure A4), the regression coefficient between future confidence intercept and interpersonal relationships was significant (*B* = 1.26, *SE* = 0.24, *p* < 0.001), suggesting that adolescents with higher initial levels of future confidence also reported better-developing interpersonal relationships. In the parallel growth model of life satisfaction and interpersonal relationships (see Appendix A Figure A5), the regression coefficient between life satisfaction intercept and interpersonal relationship intercept was significant (*B* = 0.78, *SE* = 0.26, *p* < 0.01), suggesting that adolescents with higher initial levels of life satisfaction also reported higher initial levels of interpersonal relationships. Taken together, these results are consistent with Hypotheses 3a–b, demonstrating significant co-development patterns between interpersonal relationships and all three indicators of temporal psychological well-being.

## 4. Discussion

This study employed the LGCM model to delve into the developmental trajectory and influencing factors of Chinese adolescents’ temporal psychological well-being, which encompasses subjective well-being, future confidence, and life satisfaction. It also meticulously examined the dynamic relationship between temporal psychological well-being and interpersonal relationships in adolescents. Through three waves of consecutive measurements, we found that adolescents’ subjective well-being, future confidence, and life satisfaction all exhibited a linear downward trend. Moreover, significant individual differences in subjective well-being were observed. These findings offer a novel perspective for comprehending the development of psychological well-being in adolescents.

### 4.1. Developmental Trajectories of Adolescents’ Temporal Psychological Well-Being

Firstly, subjective well-being in adolescents showed a linear downward trend across the three measurements, consistent with other studies (Chui & Wong, 2016; González-Carrasco et al., 2017; Michel et al., 2009). According to self-determination theory, intrinsic motivation is associated with subjective well-being (Flannery, 2017). However, during this stage, adolescents face academic pressure and often lack autonomy. Their behaviors are more geared toward meeting external expectations and requirements rather than intrinsic interests and enthusiasm (W. Liu et al., 2016). In such cases, their need for autonomy is unmet, and intrinsic motivation decreases, which coincides with lower subjective well-being. For instance, high parental and teacher expectations, along with academic performance pressures, may make adolescents feel forced to study rather than study voluntarily. This is associated with a reduced sense of autonomy and intrinsic motivation, and lower subjective well-being. Additionally, the individual differences in the rate of decline in subjective well-being suggest that changes in adolescents’ subjective well-being are not a singular linear process but are the result of multiple factors acting together, including social trust, academic pressure and family atmosphere (Mei et al., 2025). Secondly, future confidence also showed a linear downward trend across the three measurements. This aligns with the optimistic expectations about the future during early adolescence. As adolescence progresses, adolescents may experience anxiety and uncertainty due to a lack of clear direction, coupled with increasing uncertainties and real-life pressures, leading to a decline in future confidence. Particularly during the college entrance examination stage, adolescents aged 16–18 often face greater academic burdens and psychological stress (Zhang et al., 2015). Furthermore, under the dual influence of the Chinese education system and cultural values (K. Liu et al., 2025), such as the “elite culture” of Confucianism (Liang, 2024) and the strong link between academic performance and future career development (Fan et al., 2024), adolescents may feel more confused about the future, thereby weakening their confidence.

Lastly, life satisfaction in adolescents exhibited a linear downward trend. During this stage, adolescents’ brains are in a period of specific maturation and vulnerability. This makes them more susceptible to a series of neurobiological and psychological events when exposed to stress (S. L. Andersen & Teicher, 2008). The decline in mental health during this period is not only associated with a higher incidence of depression but may also lead to reduced life satisfaction (Orben et al., 2022). Moreover, the stress response model may accelerate this process (Tonhajzerova & Mestanik, 2017). Therefore, the interaction between the neurodevelopmental characteristics of adolescence and environmental stress may represent a key mechanism associated with adolescents’ mental health issues and declining life satisfaction.

### 4.2. The Relationship Between Predictors and Adolescents’ Temporal Mental Well-Being

Firstly, health status demonstrated a robust association with the initial levels of adolescents’ temporal psychological well-being. Adolescents in better health reported higher levels of subjective well-being and life satisfaction. This finding aligns with previous research, indicating that good health is a crucial foundation for adolescents’ temporal psychological well-being (Nagy-Pénzes et al., 2020). Better physical health is associated with reduced stress and discomfort in life, and higher emotional and cognitive well-being. Furthermore, healthy individuals are typically more engaged in social activities, which may further boost their future confidence and life satisfaction (T. H. Kim, 2024).

Secondly, the differential impact of parental relationships deserves attention. This study found that the harmony of the mother–child relationship significantly predicted subjective well-being, while the father–child relationship played a more important role in future confidence and life satisfaction. These findings are consistent with the self-differentiation perspective of family systems theory, which posits that adolescents and parents are emotionally interconnected and responsive to each other (Rothbaum et al., 2002). Mother–child relationships are more strongly associated with daily emotional experiences, whereas father–child relationships demonstrate stronger associations with long-term goals and self-identity. This differential effect may stem from the distinct socialization roles within parental relationships. Mothers are often the primary providers of daily emotional support (Lougheed et al., 2016), while fathers are more involved in future planning and self-identity formation (Shulman, 1997). Therefore, this difference stems from the different socialization roles of parents.

Thirdly, the significant negative association of academic pressure suggests its complex link to adolescents’ psychological development. This result suggests that academic pressure is associated with adolescents’ initial levels of happiness and a steeper decline in future confidence. Academic pressure is associated with reduced adolescent autonomy and competence—core needs in self-determination theory—and lower their subjective well-being (Ryan & Deci, 2000). Additionally, the high academic expectations and over-intervention of Chinese parents are associated with adolescents’ psychological burden and lower their happiness levels, a finding consistent with previous research (Chao, 1994). Some scholars conceptualize this phenomenon as “Confucian meritocracy”, in which academic achievement is regarded as the core path to social mobility and family honor (Liang, 2024). Under such cultural norms, adolescents gradually form the perception that their personal future and self-worth are closely tied to academic examination results. This prevailing meritocratic mindset becomes especially prominent during the Gaokao preparation period among adolescents aged 16 to 18. Paradoxically, excessive emphasis on academic success is associated with elevated psychological pressure attached to school performance, as well as academic anxiety and lower their future confidence instead of boosting learning motivation.

Fourthly, gender differences in adolescents’ future confidence were significant, with male adolescents exhibiting higher initial levels. This may relate to social role theory (Archer, 1996), which posits that cultural expectations of gender roles endow males with stronger self-efficacy and future control. Furthermore, biopsychological research suggests that gender hormone differences may influence the development of optimism (E. Andersen et al., 2024).

### 4.3. Interpersonal Relationships and the Developmental Changes in Adolescents’ Subjective Well-Being, Future Confidence and Life Satisfaction

Firstly, interpersonal relationships are significantly associated with subjective well-being, future confidence, and life satisfaction. This finding is consistent with previous research (Aldam et al., 2019; Bond et al., 2007; Long et al., 2021; Moore et al., 2018), suggesting that good interpersonal relationships co-occur with emotional support and resources and a higher ability to cope with life’s challenges. Furthermore, this effect may involve psychological mechanisms of belongingness and identity. For instance, friendship support can update negative self-perceptions, enhancing adolescents’ resilience and promoting the development of more positive self-views (van Harmelen et al., 2017). Secondly, this study also found associations between subjective well-being, future confidence and life satisfaction and interpersonal relationships. This suggests that higher levels of psychological well-being co-occur with better quality of interpersonal interactions. Adolescents with higher subjective well-being, future confidence, and life satisfaction tend to display more positive emotional states and behavioral tendencies and report stable social relationships. This is consistent with the “Broaden-and-Build Theory of Positive Emotions” (Fredrickson, 2001), further suggesting an association between temporal psychological well-being and interpersonal relationships.

### 4.4. Study Limitations and Prospects

First, single-item measures for subjective well-being, future confidence, and life satisfaction may not fully capture these multidimensional constructs. Adolescents may interpret future confidence in relation to specific life domains (e.g., academic or career), and responses may be influenced by transient events. Future research should employ multi-item scales or temporal qualifiers. Second, the sample of 568 adolescents, though adequate for LGCM, represents a limited proportion of the broader population. Third, social support was not examined despite its protective role in adolescent mental health. Future research should incorporate family, peer, and teacher support to examine their potential mediating or moderating effects. Fourth, the three-wave longitudinal design with two-year intervals, while appropriate for examining developmental trajectories, provides limited temporal resolution for understanding the micro-dynamics of temporal psychological well-being. Future research should consider implementing Ecological Momentary Assessment (EMA) methodologies.

## 5. Conclusions

This research found that indicators of temporal psychological well-being decline linearly between ages 16 and 19. This echoes the academic pressure, mental health challenges, and socio-cultural impacts faced by adolescents, highlighting the vulnerability of psychological well-being in this stage. Health status, academic pressure, and parent–child relationships are significantly associated with adolescents’ temporal psychological well-being. Gender also plays a role: boys start with higher future confidence than girls. Academic pressure shows a significant negative association with the initial levels of life satisfaction and the growth rate of future confidence. There is a significant co-development pattern between interpersonal relationships and psychological well-being. Good interpersonal relationships are associated with higher adolescent subjective well-being, future confidence, and life satisfaction. Similarly, higher psychological well-being is associated with better quality of interpersonal relationships. In light of these findings, comprehensive interventions should be implemented to boost adolescents’ psychological well-being and promote their healthy development. Grounded in Bronfenbrenner’s bioecological model, our findings inform multi-level interventions. At the family level, mother-focused emotional support programs may enhance subjective well-being, whereas father involvement in future planning may strengthen future confidence and life satisfaction—reflecting the distinct parental socialization roles identified in our conditional models. At the school level, curriculum reform reducing exam-oriented pressure, coupled with mental health education and peer support, may buffer academic pressure on both the initial levels and developmental trajectories, particularly for students facing the Gaokao. Given the declining trajectories across all indicators, routine well-being screening in secondary schools is warranted. At the individual level, integrating physical and mental health services is essential, as health status consistently predicted all well-being dimensions. Social skills training may yield reciprocal benefits for interpersonal relationships and psychological well-being through the co-development mechanisms identified in our parallel growth models.

## Figures and Tables

**Table 1:** A linear unconditional model of temporal psychological well-being and interpersonal relationships.

Variable	χ^2^	df	CFI	TLI	RMSEA	SRMR	Means		Variances		Intercept-Slope Covariance
							I	S	I	S	
1	1.09	1	1.00	1.00	0.01	0.01	7.93 ***	−0.12 **	1.60 ***	0.25 **	−0.36 **
2	0.00	1	1.00	1.02	0.00	0.00	4.04 ***	−0.08 ***	0.18 ***	0.02	0.00
3	3.23	1	0.99	0.96	0.06	0.02	3.94 ***	−0.07 **	0.16 **	0.04	0.00
4	0.01	1	1.00	1.01	0.00	0.00	6.99 ***	−0.02	0.81 ***	0.05	−0.01

Note: Variable 1 is subjective well-being; Variable 2 is future confidence; Variable 3 is life satisfaction; Variable 4 is interpersonal relationship. I = intercept; S = slope. Covariance refers to the unstandardized covariance between the intercept and slope factors. ** *p* < 0.01, *** *p* < 0.001.

## Data Availability

The data presented in this study are available on request from the corresponding author.

## Appendix A

**Table A1:** Descriptive characteristics of the study sample.

Variable	Category	Frequency	Percentage (%)
Age	16	170	29.9
	17	120	21.1
	18	151	26.6
	19	127	22.4
Gender	Male	283	49.8
	Female	285	50.2
Education Level	Junior High School	84	14.8
	Senior High School	382	67.3
	Vocational college	59	10.4
	Undergraduate	43	7.5
Total		568	100.0

**Table A2:** Fit indices for conditional models of subjective well-being, future confidence, and life satisfaction.

Model	χ^2^	df	CFI	TLI	RMSEA	SRMR
1	12.27	7	0.99	0.96	0.04	0.02
2	4.97	7	1.00	1.03	0.00	0.01
3	8.11	7	1.00	0.98	0.02	0.02

Note: Model 1 is the conditional model for subjective well-being; Model 2 is the conditional model for future confidence; Model 3 is the conditional model for life satisfaction.

**Table A3:** Fit indices for parallel growth models.

Model	χ^2^	df	CFI	RMSEA	SRMR
1	100.02	20	0.92	0.08	0.03
2	37.82	20	0.97	0.04	0.02
3	29.67	20	0.98	0.03	0.03
4	91.12	20	0.92	0.08	0.03
5	35.58	20	0.97	0.04	0.02
6	30.03	20	0.98	0.03	0.03

Note: Model 1 is interpersonal relationships and subjective well-being; Model 2 is interpersonal relationships and future confidence; Model 3 is interpersonal relationships and life satisfaction; Model 4 is subjective well-being and interpersonal relationships; Model 5 is future confidence and interpersonal relationships; and Model 6 is life satisfaction and interpersonal relationships.
